# Influencing the Size and Shape of High-Energy Ball Milled Particle Reinforced Aluminum Alloy Powder

**DOI:** 10.3390/ma15093022

**Published:** 2022-04-21

**Authors:** Maik Trautmann, Husam Ahmad, Guntram Wagner

**Affiliations:** Professorship of Composites and Material Compounds, Chemnitz University of Technology, 09107 Chemnitz, Germany; husam.ahmad@mb.tu-chemnitz.de (H.A.); guntram.wagner@mb.tu-chemnitz.de (G.W.)

**Keywords:** metal matrix composites, spheroidization, composite powder, additive manufacturing

## Abstract

High-energy ball milling represents an efficient process for producing composite powders consisting of ceramic particles dispersed in a metallic matrix. However, collision events, plastic deformations, and cold welding during the milling lead to a flake or block-like shape of the resulting composite powders. Further consolidation of such irregularly shaped powders by powder bed-based additive manufacturing technologies can be challenging because of their low flowability and low bulk density. In this work, different approaches, including milling process parameters (speed, process control agent atmosphere) and post-treatments (mechanical and thermal), are investigated on their suitability to influence the particle shape, especially concerning the roundness of the composite powders consisting of the aluminum alloy AlSi10Mg with 5 vol% SiC and Al_2_O_3_ reinforcement. It is found that milling with menthol as a process control agent leads to the finest composite powder compared to other milling parameters, with the lowest particle roundness of 0.39 (initial powders 0.84). No success in rounding the milled composite powder could be achieved through mechanical post-treatment in a planetary ball mill. On the other side, the thermal spraying of, e.g., SiC reinforced AlSi10Mg powder resulted in a 77–82% relative roundness. A remarkable change in the microstructure and the shape of the composite powders could also be observed after heat treatment in tube furnaces at a temperature above the melting point of AlSi10Mg. The best result in terms of improved roundness (relative to around 85%) was obtained for Al_2_O_3_ reinforced at 600 °C. A further increase of the temperature to 700 °C resulted in a moderate coarsening of powders with Al_2_O_3_ and extensive sintering of powders with SiC, presumably due to a different distribution inside the matrix.

## 1. Introduction

Aluminum and aluminum alloys belonging to a material category have widespread applications in many civil sectors, automobile, and aerospace industries, among others, not only due to their low density and high specific strength, good machinability, and relatively low cost. Further improving and adjusting of the properties of aluminum alloys can be achieved by incorporating an additional reinforcing hard phase into the microstructure. With respect to low-cost production, particle reinforcement represents a simple and effective way to improve the properties of aluminum alloys. This is because of their ability to be processed with traditional and established technologies used for monolithic materials. Thus, particle reinforced aluminum matrix composites (AMC) are produced by powder metallurgy methods and melt metallurgy methods [1,2].

One of the main strengths of the powder metallurgy route is the possibility of dispersing nano scaled ceramic particles within the metallic matrix through—for example—high-energy ball milling (HEBM), also known as mechanical alloying (MA). The resulting composite powder is then compacted by means of one of the conventional press techniques such as axial pressing or isostatic pressing. Using this approach, up to 15 vol% SiC particle fraction could be homogeneously distributed in the aluminum alloy AA2017, which significantly improved yield strength and tensile strength by about 96% and 60%, respectively [3,4]. However, this increase in the tensile properties is accompanied by an increase in hardness and wear resistance, making the final processing of AMCs, especially by machining, highly challenging and expensive due to the intensive abrasive behavior of the ceramic reinforcements [5]. In order to overcome the geometry restriction by conventional methods and minimize the post-processing of AMCs, additive manufacturing technologies such as powder bed fusion (PBF) are increasingly considered an attractive processing option for AMC parts. PBF’s capability for realizing such particle reinforced aluminum alloys was demonstrated for e.g., in TiC_p_/AlSi10Mg [6], SiC_p_/AlSi12 [7], TiB_2_/AlSi10Mg [8], TiN/AlSiMg [9], SiC/AlSi10Mg [10].

Compared to traditional powder compacting methods, the powder characteristics like particle shape, particle size, apparent density, and powder flowability play a more decisive role in the successful production of AMC parts. In this context, an investigation by [11] revealed that powders with smaller particle size distribution could be easily melted and yielded high density, high mechanical strength, and productivity. Furthermore, the powder particles’ spherical morphology and smooth surface resulted in good flowability and homogeneous layer distribution [12]. In the case of additive manufacturing of parts from pure elements or alloy, a powder preparation step is necessary when producing particle reinforced composites. In the literature, the individual powders (reinforcement and matrix) were mixed together prior to the additive manufacturing process by using a Turbula^®^ shaker (Willy A. Bachofen AG, Muttenz, Switzerland) or planetary mill. The parameters used to prepare the mixtures were carefully determined to maintain the spherical form of the powder in order to obtain the high flowability of the mixed powders during the manufacturing process. Moreover, it aimed at achieving uniform dispersion of the reinforcing components on the surface of the particles of the Al-powder or in the gaps between them [13,14]. However, to exploit the full potential of a particle reinforcement, especially for submicron particle dimensions, it is important to achieve an even particle distribution within the matrix that is not realizable by powder mixing. Thus, dispersing the reinforcing phase within the matrix in the powder preparation step could benefit the additive manufactured AMC parts. HEBM is an established method to produce composite powders from individual components with adequate homogeneity, even for nanoscale reinforcements of aluminum [15,16] and magnesium [17,18,19]. As the process consists of several mechanisms, e.g., particle deformation, particle breaking, and cold welding, the production of composite powders is accompanied by a substantial change in the powder size and morphology, which can negatively affect the powder processing by means of additive manufacturing. Therefore, this article addresses the aspects of powder morphology and size during the production of composite powders consisting of AlSi10Mg alloy reinforced with SiC and Al_2_O_3_ reinforcements. In addition, it also considers different approaches to influence the morphology of HEBM-powders for potential use as starting materials in additive manufacturing to produce particle reinforced AMCs.

## 2. Materials and Methods

### 2.1. Powders

**Figure 1:**
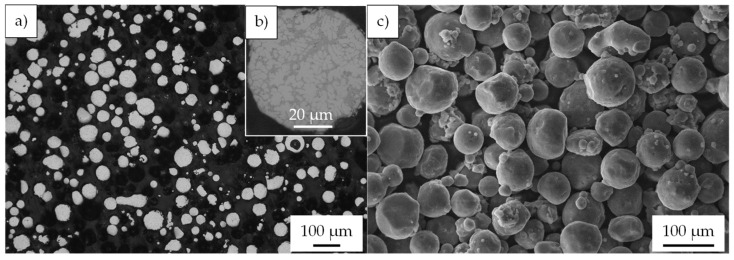
Gas-atomized Al-alloy powder AlSi10Mg0.4: (**a**,**b**) light microscopic images of the cross-section of the powder; (**c**) SE-SEM image.

The powders used for the milling experiments are nitrogen-atomized Al-alloy AlSi10Mg0.4 (TLS Technik GmbH, Bitterfeld-Wolfen, Germany) with d10 = 28 µm, d50 = 43 µm, and d90 = 93 µm. The chemical composition of the Al-alloy is listed in Table 1, and microscopic images are shown in Figure 1. As reinforcements, alpha Silicon carbide (SiC_p_) (ESK-SiC GmbH, Frechen, Germany) with d10 = 0.15 µm, d50 = 0.7 µm and d90 = 1.4 µm and alumina powder (Al_2_O_3p_) with d10 = 0.25 µm, d50 = 0.37 µm, d90 = 1.12 µm (Alfa Aesar, Kandel, Germany) were used. The aim was to study two composite powders with the same volume content of particles but different reaction behavior in the aluminum melt.

(i) AlSi10Mg + 5 vol% SiC_p_
(ii) AlSi10Mg + 5 vol% Al_2_O_3p_

### 2.2. Powder Processing

The production of composite powders was conducted in the high-energy ball mill Simoloyer CM08 (Zoz GmbH, Freudenberg, Germany) with a tank volume of 5 L that uses milling balls of stainless steel G600 (X46Cr13) with a diameter of 4.762 mm. The used ball to powder ratio was 10:1. Prior to the mechanical alloying, the individual powders were homogenized in a tumble mixer for 15 min. To study the effect of different milling parameters on the particle morphology, reference composite powders were also produced with the milling parameters listed in Table 2, which is referred to as the Reference program. Three other programs with varied parameters such as milling speed, process control agent (PCA), and milling atmosphere were considered for the purpose of higher composite powder roundness. Compared to the reference program, the period of the high rotation speed (600 rpm) was split in Program1, and a lower milling speed of 200 rpm was implemented. Higher milling speed involves higher impact force on the mill charge and this, in turn, promotes the cold welding resulting in a rapid powder coarsening. Lowering the milling speed in the last speed sequence should otherwise promote breaking up the lamellar structure resulting from the cold-welding process to equiaxial particle form.

In Program2, the speed was kept unchanged, and the PCA stearic acid was replaced by menthol, which is proven to be a suitable alternative to stearic acid and has a higher refining effect concerning the particle size [20]. Due to its lower melting point compared to stearic acid (Table 3), it minimized the cold welding and the adhesion of the powder to each other and the rotor, grinding chamber, and grinding balls. The comparison shows that stearic acid can withstand higher temperatures. Since the temperature in the collision points between the grinding balls is significantly higher than the process temperature, both agents melt and coat the rotor, grinding balls, and powder grains.

**Figure 2:**
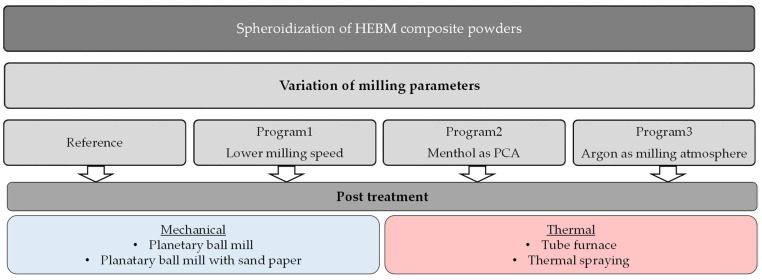
Overview of the different approaches used to influence the morphology of the composite powders.

In addition, each of the composite powders realized by the different milling programs was subjected to a post-treatment process to change the powder morphology towards a spherical form. An overview of the adopted approaches for spheroidization of the composite powders is given in Figure 2. Basically, the post-treatment can be divided into two main categories: mechanical treatments and thermal treatments.

The mechanical post-treatment in planetary ball mill Fritsch Pulverisette 5 (Fritsch GmbH, Idar-Oberstein, Germany) consisted of two experiments with the same milling parameters (150 rpm for 1 h in air atmosphere). In the first experiment, the HEBM-powder was milled with a ball to powder ratio of 5:1 with the mentioned lower milling speed aiming to break up the lamellar towards more homogeneous particle size and more roundness. In the second configuration, the milling balls were omitted, and the inner wall of the grinding jar was equipped with sandpaper with a milling program-dependent grit size (120 rpm for Reference and Program1, 240 rpm for Program2, and 16 rpm for Program3). This was aimed to round the composite powder through abrasion by a relative rubbing against the sandpaper.

The thermal treatment in the tube furnace covered a temperature range from 515 °C (below the melting point of 576 °C of the AlSi10Mg alloy) up to 700 °C. In one experiment, all composite powders based on SiC reinforcement with different milling programs were kept at 515 °C for a holding time of 3.5 h. Further experiments included the thermal treatment of 5 vol% SiC or 5 vol% Al_2_O_3_ reinforced composite powders (both milled with 0.0625 m%/h Menthol for 7 h) at temperatures of 600 °C, 650 °C, and 700 °C for a short holding time of 1 min to study the impact of the occurring melting phase on the powder shaping. From preliminary tests, it is known that particle reinforced aluminum powders have a lower tendency to pressureless sintering than the unreinforced alloy. Even at temperatures above the melting point of the matrix alloy, the composite powders retain their solid shape. This, in addition to the short holding time of 1 min, is the reason behind choosing a temperature above the melting point of the matrix.

**Figure 3:**
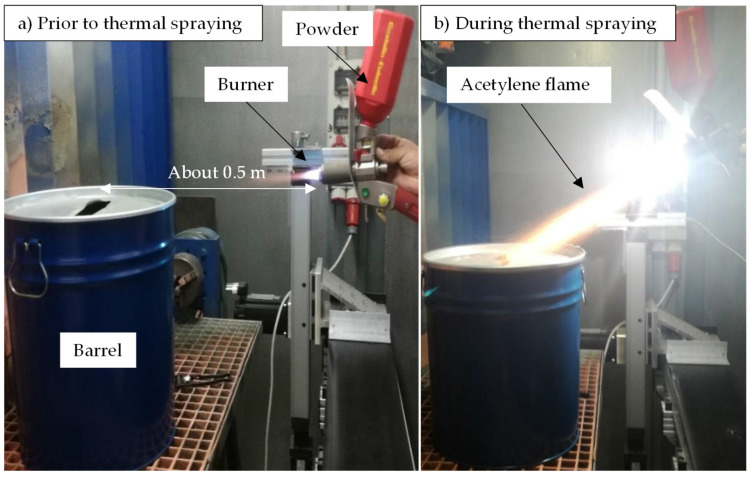
Experimental setup of thermal spraying: (**a**) prior and (**b**) during powder treatment.

The second variant of the thermal post-treatment included a thermal spraying process (GTV TopGun G acetylene burner) of the HEBM-powder through a spray gun using an acetylene/oxygen mixture at a temperature of 2600 °C. The total experiment time was about 1 min for a powder amount of 150 g. The treated powder is then caught by a barrel positioned at about 0.5 m from the spray device. The experimental setup is demonstrated in Figure 3. To avoid a possible blocking of the spray gun during the feeding phase, the HEBM-powder was sieved with a 70 µm sieve to remove coarse and oversized particles.

### 2.3. Powder Characterization

**Figure 4:**
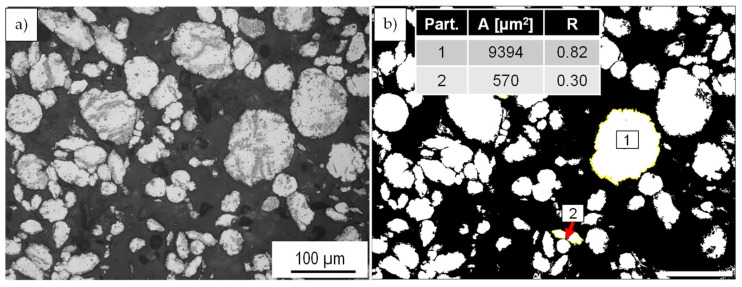
Analysis of the particle shape and particle area: (**a**) original light microscopic cross-section image and (**b**) processed picture with the image processing software ImageJ.

The analyses of the composite powders regarding their size and shape were carried out on cross-sections of the composite powders. For this purpose, images obtained from an inverse light microscope OLYMPUS GX51 (OLYMPUS EUROPA SE & CO. KG, Hamburg, Germany) were further processed by an image analyzing software ImageJ [23] to detect the particle size and morphology change depending on the varied milling parameters and post-treatment. An example of an original (Figure 4a) and an analyzed picture is shown in Figure 4b. According to the software algorithm, it can be distinguished between two form factors. One is the circularity of the powder particles with the Equation (1):

Circularity = 4π × [Area]/[Perimeter]^2^ (1)

Equation (2) describes the roundness, which can be calculated by

Roundness (or the inverse of Aspect Ratio) = 4 × [Area]/(π × [Major axis]^2^) (2)

A calculation of different particle shapes with a comparable particle (Table 4) demonstrates the main difference between these two form factors. A perfect circular particle as Particle 1 has a circularity and roundness of 1. In comparison, Particle 2 represents the case of a round shape with a kind of clefts and pronounced uneven, rough surface. This still results in high roundness values of 0.98 while the circularity sharply drops to 0.33 due to a larger perimeter. Even in the case of elongated particles (Particle 3), the roundness form factors give a more appropriate description of the particle shape. Therefore, the roundness is mainly used to consider the change in the particle shape of the milled powder. Another aspect of using the roundness is that the treated composite powders possess a rough surface, and thus a precise determination of the perimeter is not possible. The use of perimeter could lead to smaller circularity values, while the roundness value remains less sensitive to this kind of artifact. The mean values of particle area and roundness were calculated from three images of the representative area of the cross-section.

The samples were further analyzed by scanning electron microscopy (SEM, Zeiss Leo1455VP, Jena, Germany) to obtain more information about the phase composition and possible reaction. The microscope is equipped with a secondary-electron detector (SE), a backscattered-electron detector (BSE), and energy-dispersive X-ray microanalysis (EDXS).

Untreated AlSi10Mg powder has been considered a reference to assess the effect of the chosen approach on the roundness of the composite powders. Particles that were too close or particle agglomerations in the powder cross-sections were excluded from the calculations.

## 3. Results

### 3.1. Influence of Milling Parameters

**Figure 5:**
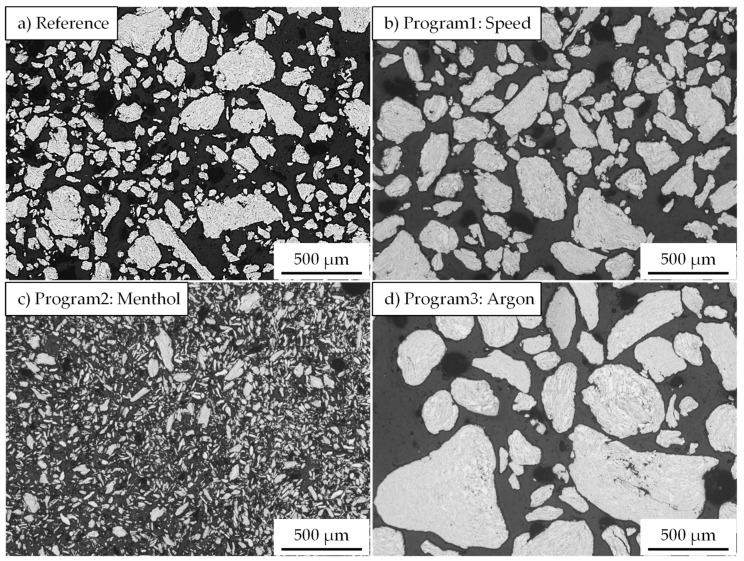
Influence of different milling programs (**a**) Reference, (**b**) Program1 with speed change, (**c**) Porgramm2 with menthol as PCA, and (**d**) Program3 with argon atmosphere on the size and the shape of the HEBM composite powder.

**Figure 6:**
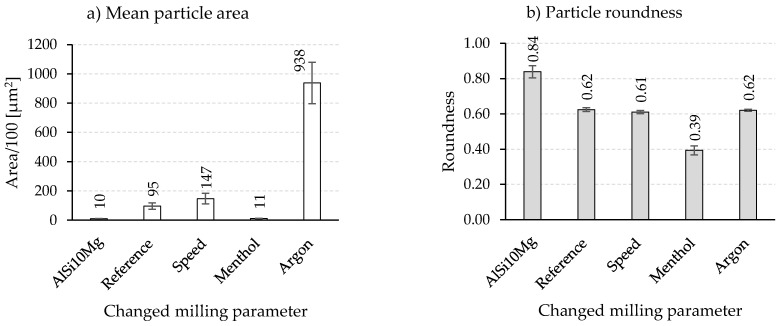
Results of (**a**) mean particle area and (**b**) roundness of composite powders produced with different milling parameters.

An overview of the composite powders produced by the different milling programs is given in Figure 5. The light microscopic images show a pronounced effect of the milling parameters on the particle size. Compared to the initial spherical powder, a significant change in the particle morphology can be observed for all milling programs. However, differences exist among the different programs, especially regarding the particle size and particle size distribution. The integration of low speed in Program1 leads, with respect to the Reference program (Figure 5a), to an increase in particle size and better particle size distribution (Figure 5b). Thus, a higher mean particle area of 14,700 µm^2^ (9500 µm^2^ for the Reference program) is measured (Figure 6a). Nevertheless, the roundness of the composite powder remains unchanged and stays similar to the Reference program with a value of 0.61. Oppositely, using menthol in Program2 instead of stearic acid results in completely different composite powder characteristics. The light microscopic image in Figure 5c shows the pronounced reduction in the particle size and area (1300 µm^2^), which is considerably lower than that of the Reference program and comparable to that of the initial powder of the aluminum alloy (1000 µm^2^) (Figure 6a). The PCAs are absorbed on the surface and act as a separating layer that changes the cold welding effect.

It could be assumed that the lower melting temperature and smaller molecular size of menthol provide better surface wetting compared to stearic acid. This indicates a profound effect of menthol, which prevents cold welding of the powder and leads to more intensive work hardening and breaking events during the milling process. Nonetheless, the flattened or elliptical powder form in the case of menthol dominates, and thus the powder roundness is reduced to about 0.39 (Figure 6b).

An opposite trend during the forming of composite powder is detected when replacing rest air in the milling chamber with a constant flow of argon. As can be seen in Figure 5d, milling under an argon atmosphere causes an obvious increase in the particle size so that the mean particle area changes dramatically to a high value of 93,800 µm^2^ (Figure 5a). The main increase in the particle size is noticed to accelerate from a milling time of 3 h. This was also accompanied by a simultaneous roundness of the composite powders. The comparative value of the reference sample of 0.62 was reached.

### 3.2. Influence of Post Treatment

#### 3.2.1. Planetary Ball Milling (PBM)

**Figure 7:**
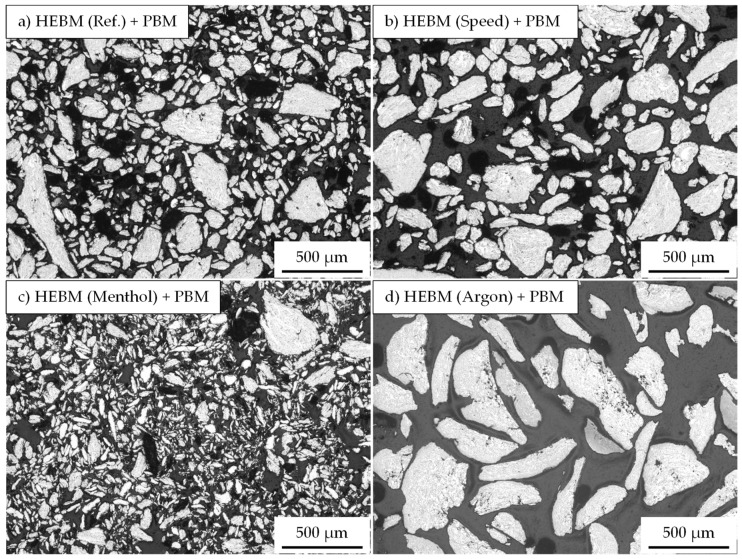
Light microscopic images of the HEBM composite powder: (**a**) Reference, (**b**) variation of milling speed (Program1), (**c**) milled with menthol (Program2), and (**d**) milled in argon atmosphere (Program3) after a mechanical post-treatment in a planetary ball mill (PBM).

**Figure 8:**
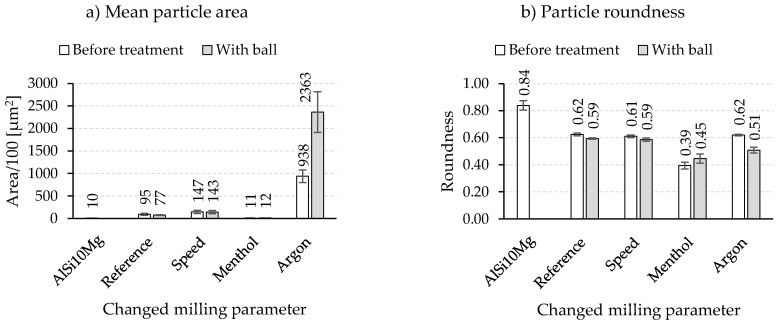
Results of (**a**) mean particle area and (**b**) roundness after a mechanical post-treatment in planetary ball mill.

The light microscopy results, the mean particle area, and the roundness of composite powders after mechanical post-treatment are summarized in Figure 7 and Figure 8, respectively. Further milling of the reference composite powder in a planetary ball mill (with balls) did not cause any noteworthy change in both the particle area (7700 ± 1000 µm^2^ vs. 9500 ± 2200 µm^2^ for untreated powder) and the roundness (0.59 ± 0.0 vs. 0.62 ± 0.1 for untreated powder). The HEBM composite powders produced with modified speed sequences and a different process control agent (menthol) also showed similar behavior. Conversely, the HEBM powders milled under an argon atmosphere experienced an increase in the particle area accompanied by a decline in the particle roundness from 0.62 to 0.51, which was in contrast to the expected breaking effect of the lower speed applied in the planetary ball mill.

No noteworthy change in particle size could be reported in the second milling experiment carried out with the sandpaper (without milling balls). Additionally, no improvement of the roundness could be reached with this approach (data not shown).

#### 3.2.2. Tube Furnace

**Figure 9:**
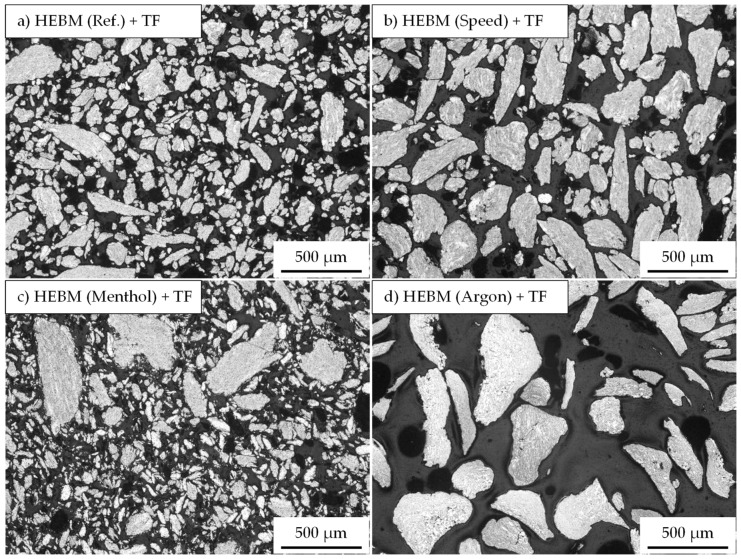
Light microscopic images of the HEBM composite powders produced by (**a**) Reference, (**b**) Program1 (Speed), (**c**) Porgram2 (Menthol), and (**d**) Porgram3 (Argon) after a thermal post-treatment in a tube furnace (TF) at 515 °C for 3.5 h.

**Figure 10:**
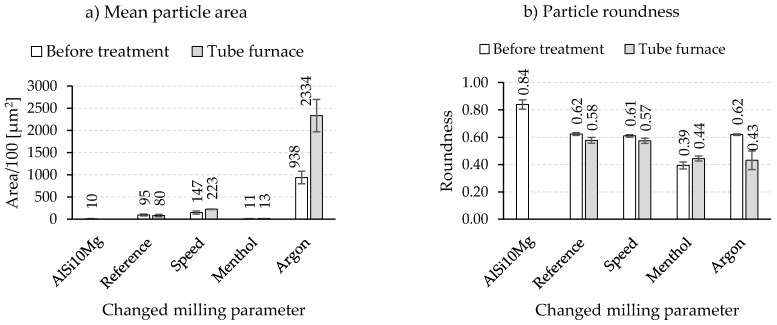
Results of (**a**) mean particle area and (**b**) roundness after a thermal post-treatment in a tube furnace at 515 °C for 3.5 h.

The light microscopic images in Figure 9 reveal that a thermal treatment, even at a temperature near the aluminum alloy’s melting point, does not affect the roundness of the composite powders. Measurements of the particle area and roundness of the heat-treated powders confirm this observation (Figure 10). Similar to the mechanical post-treatment, the change in the roundness is very small and negligible. Only for the powders milled in an argon atmosphere, a higher mean particle area of 233,400 µm^2^ and a lower roundness value of 0.43 with a larger standard deviation are calculated.

**Figure 11:**
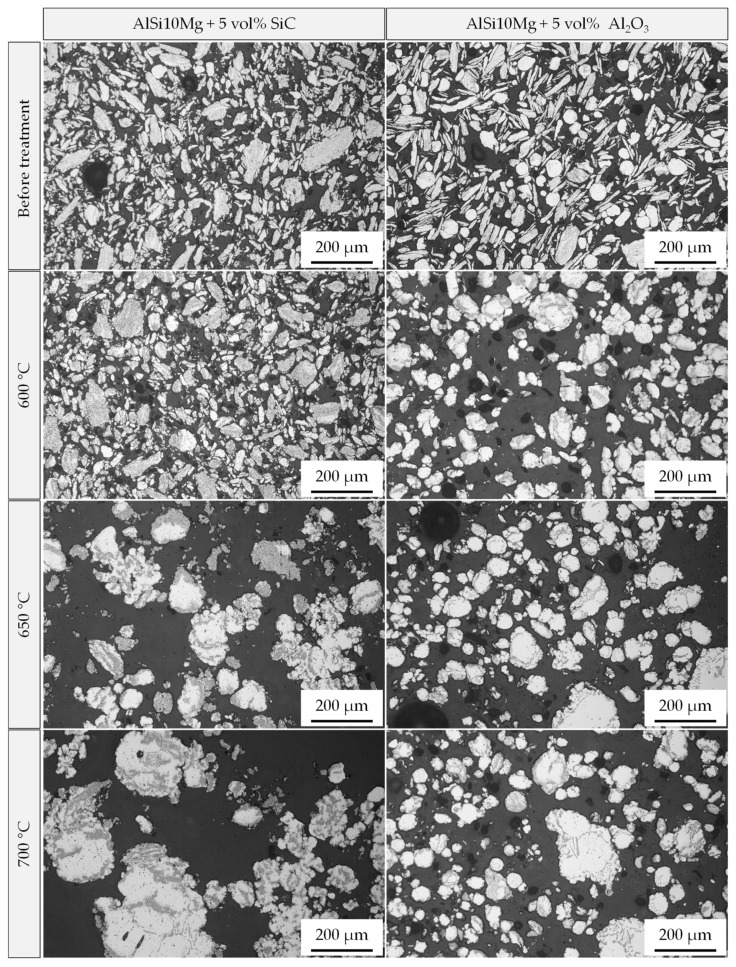
Light microscopic images of the composite powders after a thermal post-treatment in a tube furnace at different temperatures for 1 min above the melting point of the AlSi10Mg alloy for 5 vol% SiC and Al_2_O_3_ reinforcement (composite powders produced with menthol as PCA).

**Figure 12:**
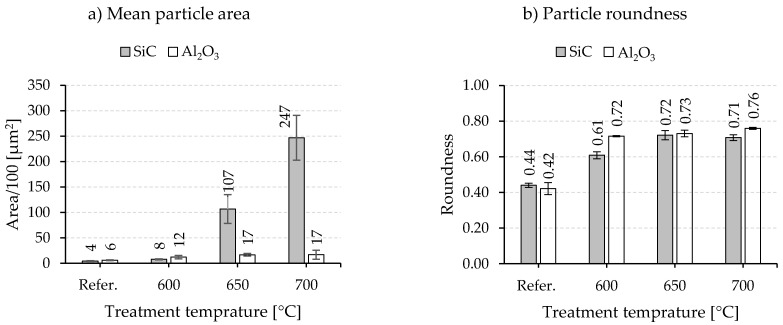
Mean particle area (**a**) and roundness (**b**) after a thermal post-treatment in a tube furnace at different temperatures for 1 min above the melting point of the AlSi10Mg alloy for 5 vol% SiC and Al_2_O_3_ reinforcement (composite powders produced with menthol as PCA).

In comparison, a remarkable change in the powder morphology is evidenced after heat treatment above the alloy’s melting temperature. The light microscopic images in Figure 11 demonstrate the response of composite powders reinforced with 5 vol% SiC and 5 vol% Al_2_O_3_ (both milled with menthol) to the different treatment temperatures and the occurrence of a liquid phase. In the case of SiC reinforced composite powder, the treatment at 600 °C causes a limited change in the powder shape. This becomes more obvious after raising the temperature to 650 °C and 700 °C, whereby a simultaneous powder coarsening due to particle sintering and the appearance of an unreinforced area of the Al-alloy is observed (Figure 11). Measurements of the mean particle area and the particle roundness also reflect the observed change in size and shape (Figure 12a,b), especially after the heat treatment at 650 °C and 700 °C with a significant increase in the mean particle area from 400 µm^2^ (untreated) to 10,700 µm^2^ and 24,700 µm^2^, respectively. At the same time, the roundness improves to 0.61–0.72 in the considered temperature range. Al_2_O_3_ reinforced composite powders, on the contrary, show a different behavior during the heat treatment under the same temperatures. As can be seen from Figure 11, the rounding of the particles takes place even at 600 °C, and the particle coarsening and sintering remains to a limited extent. This is also shown in Figure 12. The mean particle area changes between 1200 µm^2^ and 1700 µm^2^ when treated between 600 °C and 700 °C. The roundness reaches a value of 0.72 at 600 °C (compared to 0.42 before treatment) with a slight increase with rising temperatures (Figure 12b). It should be mentioned here that considering mean particle area aims to express the relative development of the particle size. It is not the aim to measure the real particle size.

#### 3.2.3. Thermal Spraying

**Figure 13:**
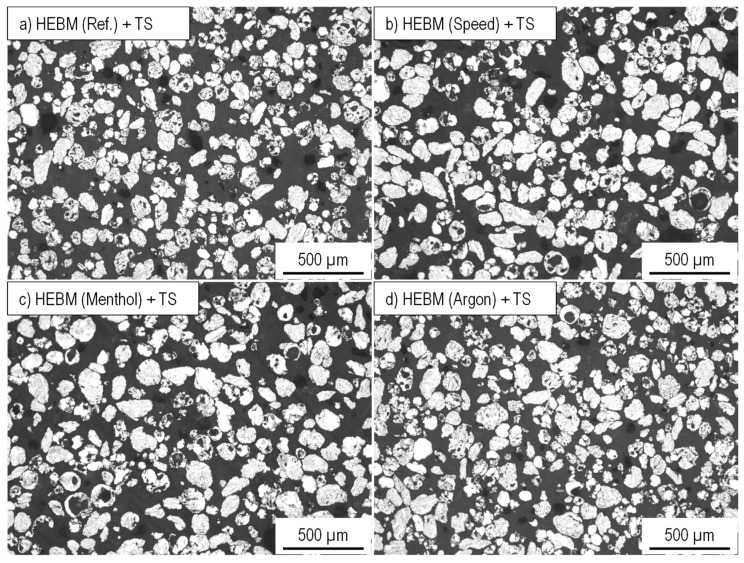
Thermal post-treatment by means of thermal spraying of HEBM-powder produced by (**a**) Reference Program, (**b**) Program1, (**c**) Program2 and (**d**) Program3.

In the case of thermal spraying, the powder was sieved with a 70 µm sieve prior to the treatment to avoid clogging the gun. Thus, only roundness is considered. Due to the high process temperature, an obvious change in the particle morphology towards more rounded particles is observed (Figure 13). In addition, the powder cross-section depicts that, apart from the milling program used, the treated powders contain a certain number of hollow particles (particles with cavities emerged during the process).

**Figure 14:**
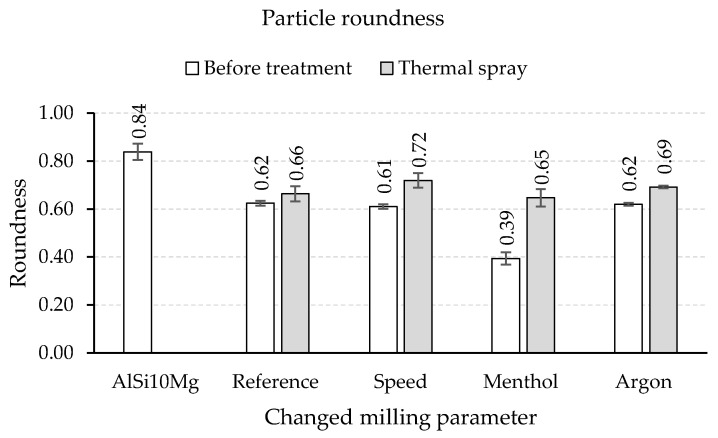
Particle roundness of HEBM-powder treated by means of thermal sprayed HEBM-powder.

The results of the corresponding image analyses of the cross-sections are summarized in Figure 14. An increase in the roundness is noticed for all composite powders produced by different milling programs. The most significant improvement is determined for the powder milled with menthol. In this case, the roundness increased by about 64% compared to other milling programs with 6% for the reference, 17% for speed, and 12% for argon.

### 3.3. Reaction and Distribution of SiC in Thermally Treated Composite Powder

The reaction and distribution of the reinforcement are essential, especially when rounding the composite powders is accomplished by applying thermal energy as in the case of tube furnace and thermal spraying.

**Figure 15:**
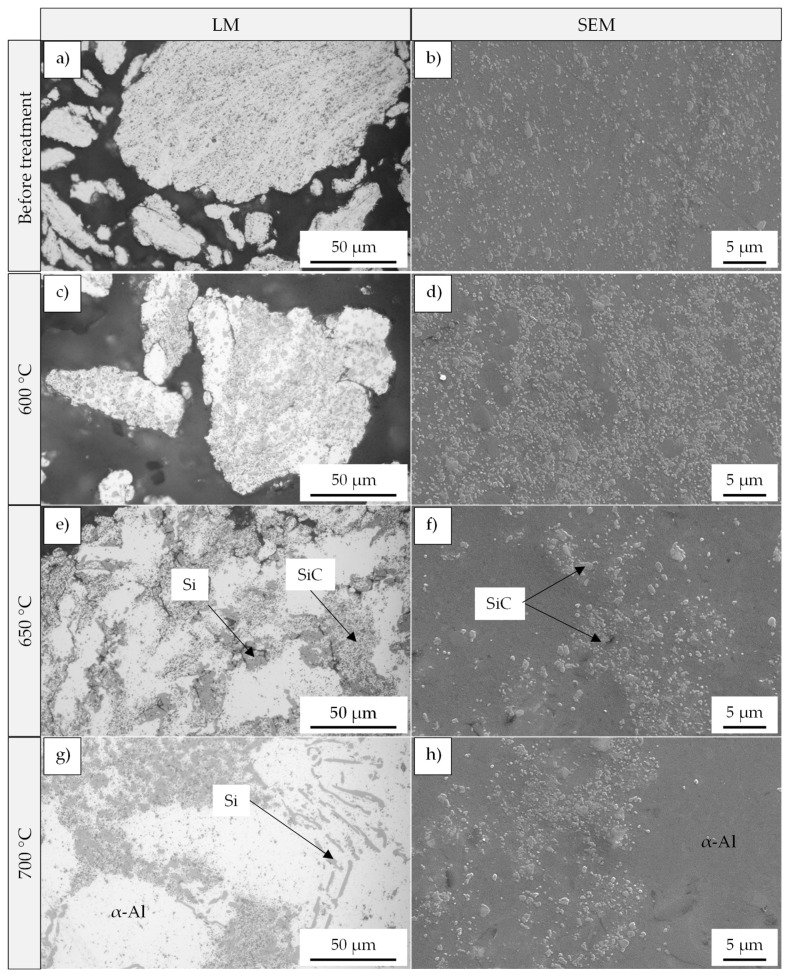
Heat treatment in tube furnace at different temperatures of composite powder (AlSi10Mg + 5 vol% SiC) milled with menthol. (**a**,**c**,**e**,**g**) light microscopic; (**b**,**d**,**f**,**h**) SE-SEM micrographs.

Since SiC is more probably to react with the Al-alloy than Al_2_O_3_, the temperature-dependent change in the microstructure during the treatment in a tube furnace is demonstrated for SiC reinforced composite powders in Figure 15.

A treatment at 600 °C causes the crystallization of silicon particles and forms the initial powders’ eutectic structure with redistribution and agglomeration of SiC (Figure 15c,d). This behavior is more pronounced after the heat treatment at higher temperatures of 650 °C and 700 °C. Reinforcement-free regions appear surrounded by the Si-particles and obvious redistribution and agglomeration of SiC. EDXS-Analysis showed that these regions consist of α-Al. Furthermore, no reaction of SiC with Al can be detected under the experimental conditions.

**Figure 16:**
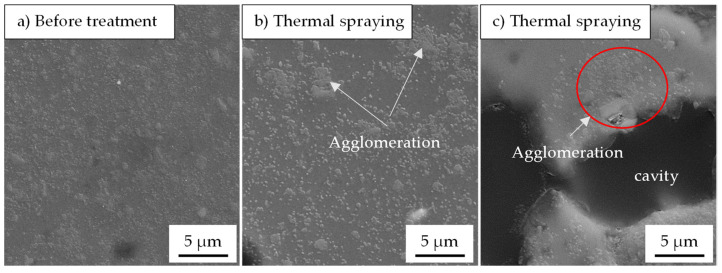
SE-SEM micrographs of the composite powders (AlSi10Mg + 5 vol% SiC) milled with menthol: (**a**) before treatment, (**b**,**c**) after thermal spraying showing the redistribution and agglomeration of SiC.

The spheroidization by means of thermal spraying also leads to redistribution and agglomeration of SiC (Figure 16). In the compact, dense particles, only a restricted or no appearance of SiC clusters is noticed (Figure 16b). On the contrary, in the hollow particles, the effect of redistribution and agglomeration is more identifiable, as shown in Figure 15c. Moreover, the crystallization of the Si-particle is not observed.

## 4. Discussion

**Figure 17:**
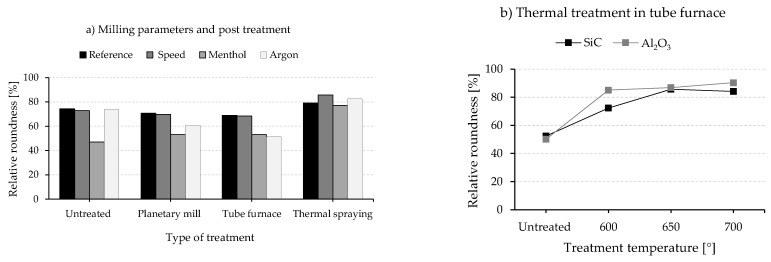
Relative roundness of composite powders produced with (**a**) different milling parameters and different post-treatments and (**b**) the influence of treatment temperature.

The milling parameters and the post-treatments modify the particle morphology and size to different extents. In this context, it is helpful to discuss the efficiency of the different approaches primarily with respect to the relative roundness compared to the as-received alloy. This is presented in Figure 17a for the different milling parameters/treatments and in Figure 17b for tube furnace experiments at different temperatures above the melting point of Al-alloy for composite powders with 5 vol% SiC and 5 vol% Al_2_O_3_ reinforcements.

The untreated powder milled with menthol possesses the lowest relative roundness of 46%. This is basically due to the inhibition of cold welding by menthol so that the composite powder is continuously deformed and broken, leading to flattened shaped and fine powders. With regard to the Reference program, the integration of a low-speed sequence of 200 rpm into the reference milling program does not affect the milling mechanisms. So both programs result in particle roundness with a relative value of 74%. Similar rounding of composite powders can be achieved using argon atmosphere, but with a significant powder coarsening due to intense cold welding, which makes this powder inappropriate for further processing in powder bed-based additive manufacturing.

The powders that were post-treated in planetary ball mill and tube furnace at 515 °C do not experience any remarkable change with respect to the particle roundness. Both the milling speed in PBM and the treatment temperature of 515 °C (below the melting point of the matrix) seem to be low to cause any particle reforming. Only after thermal treatment through thermal spraying the relative roundness improves to a value range of 77–82% for different milling parameters. During the process, the powders are subjected to a high temperature for a very short time, and thus they are heated to a molten or semi-molten state, which causes the reshaping of the particles. This can be deduced from the appearance of SiC agglomeration (Figure 16b) and hollow particles with a pronounced SiC clustering (Figure 16c). The observation of the original particle shape, rounded, and hollow particles after thermal spraying could indicate that the individual particles of the composite powder experience different process conditions in terms of exposure temperature and time. The origin of the particle cavities is not well understood yet. Still, it presumably emerges due to passing through a shortened molten state with a simultaneous escaping of the process control agent. With regard to processing with additive manufacturing, porosity formation during thermal spraying could be more disadvantageous than the occurred limited SiC agglomeration.

The treatment in a tube furnace above the melting point of the matrix can be principally compared to thermal spraying but with a longer exposure time to thermal energy in the molten state. In addition, it guarantees more homogenous heating of all particles in the treated composite powders. This approach significantly increases the relative roundness of the powder even at 600 °C to around 85% for Al_2_O_3_ and 72% for SiC reinforcements (Figure 15b). However, a further increase of the temperature to 650 °C and 700 °C leads to a relative roundness of around 85% in the case of SiC, but with remarkable sintering of the particles. This results in a coarsening of the powder and does not fulfill the requirements of powder bed-based additive manufacturing technologies of small particle sizes. On the contrary, particle coarsening of the Al_2_O_3_ reinforced composite powders remains on a lower level, which indicates higher resistance to the sintering process. This could be related to the reinforcement distribution in the milled powder prior to the thermal treatment. In general, during the milling process and under the same parameters, faster incorporation of SiC into the matrix particle than Al_2_O_3_ is observed (perhaps due to the smaller particle size of Al_2_O_3_ powder). As a result, SiC is mainly distributed within the particle volume after the milling process, while a higher amount of Al_2_O_3_ is still located on the particle surface, hindering their fusion. Treating, for example, the milled unreinforced alloy at 600 °C showed the forming of a large metallic sphere resulting from sintering, which can confirm the role of the reinforcement and their distribution on the sintering behavior. Another difference compared to thermal spraying is the obvious change in the microstructure of the composite powder after treatment in a tube furnace. Due to the longer exposure time in the molten state, the alloying elements silicon begins to crystallize. Larger Si-particles form within the α-Al phase. This is also accompanied by a migration of the reinforcement to the Si-particle-rich area leaving SiC/Al_2_O_3_ impoverished α-Al regions (Figure 15). The impact of such change in the microstructure of composite powder on further processing using additive manufacturing technologies cannot be easily derived. But it is not necessarily disadvantageous since processing for instance in powder bed fusion could redesign the microstructure regarding the matrix phase and reinforcement distribution (melting and high cooling rates).

## 5. Conclusions

High-energy ball milling (HEBM) ensures the incorporation of nanoscale reinforcements such as SiC/Al_2_O_3_ into a metallic matrix-like AlSi10Mg. It thus enables the production of composite powder, which can be further processed with additive manufacturing technologies. However, during the HEBM, the particle shape of the composite powder becomes irregular and hence deviates from the preferred spherical shape for additive manufacturing. Consequently, this can impair the powder feeding and part building. For this purpose, the influence of different approaches, including milling process parameters (speed, menthol as a process control agent, argon as milling atmosphere) and post-treatments (mechanical and thermal) on the particle shape (roundness) and coarsening were investigated. The results of the applied approaches are summarized as the following:

- **Influence of milling parameters:** Implementing a low-speed sequence into the standard milling program or using argon as a milling atmosphere has no influence on the roundness compared to the Reference program. Particles possess an irregular shape with a relative roundness of 74% (with respect to the virgin non-milled AlSi10Mg powder). In the argon case, this was also accompanied by remarkable cold welding and powder coarsening so that it can be excluded for use in additive manufacturing. Replacing stearic acid with menthol as a process control agent significantly reduces the cold welding, resulting in a very fine powder but with a lamellar shape and low relative roundness of 47%.
- **Influence of different post-treatments:** The relative roundness remained unchanged after mechanical treatment in a planetary ball mill or thermal treatment in a tube furnace at 515 °C (below the alloy’s melting temperature). On the contrary, an increase in relative roundness up to 77–82% was observed after thermal treatment by thermal spraying. However, this approach leads to the formation of hollow particles in the powder. Moreover, a partial redistribution and SiC clustering occurred, presumably due to passing through a molten state for a very short time (less than a second).
- **Influence of thermal post-treatment in tube furnace above the melting point of the alloy:** A longer thermal aging in the molten state at 600 °C, 650 °C, and 700 °C for composite powders with SiC or Al_2_O_3_ reinforcement results in an obvious change in the microstructure. This includes the precipitation of silicon particles from the original dendritic structure and the redistribution and agglomeration of the reinforcements. With regard to the relative roundness, values between 85% and 90% for temperatures in the range of 600–700 °C can be attained for Al_2_O_3_ reinforcements with a limited particle coarsening. The treatment of such composite powders above the melting point showed the best results concerning a subsequent possessing. On the other side, SiC reinforced composite powder also showed improvements in relative roundness. However, extensive powder sintering hinders further processing in additive manufacturing.

Among all investigated approaches in this article, thermal-based treatment represents the most effective and suitable way for the spheroidization of composite powders. However, the treatment temperature should be adjusted to the kind of reinforcement, its amount, and distribution. The simplest way of providing the required thermal energy is a treatment in a tube furnace with the main drawback of not complete spheroidization of all particles. To avoid this and to process larger amounts of powder, it is imaginable to circulate the powders using inert gas in a closed chamber or tubes at the appropriate process temperature. This process is already known in the industry, but for powder drying purposes. Promising is also the thermal spraying process, even though controlling the process parameters e.g., temperature could be challenging.

## Figures and Tables

**Table 1:** Chemical composition of AlSi10Mg alloy according to the datasheet of the manufacturer.

Element	Si	Mg	O	Al
**Content [m%]**	9–11	0.20–0.45	<0.4	Bal.

**Table 2:** Variation of milling parameters compared to the reference milling program.

Program	Reference	Program1	Program2	Program3
**Changed Parameter**	-	Milling speed	PCA	Atmosphere
**Milling cycle**	400 rpm for 5 min 600 rpm for 5 min	400 rpm for 5 min 600 rpm for 3.5 min 200 rpm for 1.5 min	400 rpm for 5 min 600 rpm for 5 min	400 rpm for 5 min 600 rpm for 5 min
**Milling duration**	6 h	6 h	6 h	6 h
**PCA**	Stearic acid 0.0625 m%/h	Stearic acid 0.0625 m%/h	Menthol 0.0625 m%/h	Stearic acid 0.0625 m%/h
**Milling atmosphere**	Air	Air	Air	Argon

**Table 3:** Comparison of properties between stearic acid [21] and menthol [22].

	Stearic Acid	Menthol
**Aggregate state at 20 °C**	solid	solid
**Chemical formula**	C_18_H_36_O_2_	C_10_H_20_O
**Density [g/cm^3^]**	0.94	0.89
**Melting point [°C]**	69	41–43
**Boiling point [°C]**	371	212

**Table 4:** Difference between roundness and circularity for various particle shapes.

	Particle 1	Particle 2	Particle 3
Schematic cross section	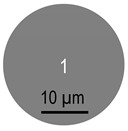	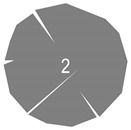	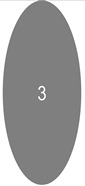
Area [µm^2^]	575	563	573
Roundness	1	0.98	0.43
Circularity	1	0.33	0.77

## Data Availability

Not applicable.
